# Ability to Gain Control Over One’s Own Brain Activity and its Relation to Spiritual Practice: A Multimodal Imaging Study

**DOI:** 10.3389/fnhum.2017.00271

**Published:** 2017-05-24

**Authors:** Silvia E. Kober, Matthias Witte, Manuel Ninaus, Karl Koschutnig, Daniel Wiesen, Gabriela Zaiser, Christa Neuper, Guilherme Wood

**Affiliations:** ^1^Department of Psychology, University of GrazGraz, Austria; ^2^BioTechMed-GrazGraz, Austria; ^3^Leibniz-Institut für WissensmedienTuebingen, Germany; ^4^LEAD Graduate School and Research Network, Eberhard Karls University TuebingenTuebingen, Germany; ^5^Division of Neuropsychology, Center of Neurology, Hertie-Institute for Clinical Brain Research, University of TuebingenTuebingen, Germany; ^6^Laboratory of Brain-Computer Interfaces, Institute for Neural Engineering, Graz University of TechnologyGraz, Austria

**Keywords:** brain volumetry, cognitive control, mental strategy, neurofeedback, prayer, spiritual practice

## Abstract

Spiritual practice, such as prayer or meditation, is associated with focusing attention on internal states and self-awareness processes. As these cognitive control mechanisms presumably are also important for neurofeedback (NF), we investigated whether people who pray frequently (*N* = 20) show a higher ability of self-control over their own brain activity compared to a control group of individuals who rarely pray (*N* = 20). All participants underwent structural magnetic resonance imaging (MRI) and one session of sensorimotor rhythm (SMR, 12–15 Hz) based NF training. Individuals who reported a high frequency of prayer showed improved NF performance compared to individuals who reported a low frequency of prayer. The individual ability to control one’s own brain activity was related to volumetric aspects of the brain. In the low frequency of prayer group, gray matter volumes in the right insula and inferior frontal gyrus were positively associated with NF performance, supporting prior findings that more general self-control networks are involved in successful NF learning. In contrast, participants who prayed regularly showed a negative association between gray matter volume in the left medial orbitofrontal cortex (Brodmann’s area (BA) 10) and NF performance. Due to their regular spiritual practice, they might have been more skillful in gating incoming information provided by the NF system and avoiding task-irrelevant thoughts.

## Background

Neurofeedback (NF) is a type of biofeedback in which one can learn to voluntarily modulate one’s own brain signals by means of real-time feedback of specific neuronal responses. Successful modulation of one’s own brain activity can lead to improvements in cognition and behavior (Gruzelier, 2014a). However, individuals greatly differ in their ability to learn during NF training. A substantial portion of potential NF users (about 15%–30%) fail to gain significant control over their own brain signals even after repeated training sessions (Allison and Neuper, 2010; Blankertz et al., 2010; Kober et al., 2015a). Some predictors/correlates of successful NF performance have been identified. Several neurophysiological predictors are relevant for successful NF performance (Neumann and Birbaumer, 2003; Kübler et al., 2004; Blankertz et al., 2010; Enriquez-Geppert et al., 2013; Halder et al., 2013a,b; Ninaus et al., 2013, 2015; Reichert et al., 2015) as well as psychological factors, such as control believes, “locus of control” (LOC), degree of concentration, mood, mastery confidence, motivation, or mental strategy used (Burde and Blankertz, 2006; Nijboer et al., 2008; Kleih et al., 2010; Hammer et al., 2012; Kober et al., 2013; Witte et al., 2013). However, the existence of a cognitive key factor for successful self-regulation of brain signals is still elusive (Wood et al., 2014).

Cognitive control and supervisory processes, such as focusing attention on internal states, self-agency or self-awareness seem to play a crucial role in NF learning (Wood et al., 2014) as revealed by prior neuroimaging studies (Ninaus et al., 2013, 2015; Emmert et al., 2016). These studies identified a network of frontal brain regions associated with NF control, which is recruited both during NF training (Emmert et al., 2016) as well as under the simple belief of receiving NF training (Ninaus et al., 2013). This network includes the insula, anterior cingulate cortex (ACC), supplementary motor and dorsomedial and lateral prefrontal areas, as well as the inferior and middle frontal gyrus (Ninaus et al., 2013, 2015; Emmert et al., 2016). These brain areas are generally associated with cognitive control processes (Ninaus et al., 2013, 2015). For instance, the insula is mainly responsible for driving attention to inner states (Barrett et al., 2004; Critchley et al., 2004; Pollatos et al., 2007) and is involved in comparing and integrating external information (e.g., provided by the feedback screen during NF training including a visual feedback modality) with internal information (e.g., sensation of own mental states, feelings, thoughts, etc. during NF training; Craig, 2009; Ninaus et al., 2013). Hence, the ability to shift attention to internal or external events (Hasenkamp et al., 2012) and to concentrate over a determined period of time seems to be relevant for NF learning.

Focusing attention on internal states and self-awareness are paradigmatic not only for NF learning but also for cognitive states during spiritual practice such as meditation, praying, or mindfulness (Moss, 2002; Lazar et al., 2005; Cahn and Polich, 2006; Farb et al., 2007; Manna et al., 2010). While focusing on oneself during spiritual practice, internal (e.g., task-irrelevant thoughts, rumination) and external disturbances have to be ignored or suppressed (Francis and Robbins, 2015). Generally, spiritual practice refers to a large variety of practices ranging from religious recitation, purely relaxation based meditation, mindfulness meditation, to meditation performed with the goal of attaining a powerful spiritual experience (Newberg et al., 2001). Beside this variation, functional brain imaging studies revealed that different spiritual practices such as Christian prayer (Azari et al., 2001), Buddhist meditation (Newberg et al., 2001, 2003; Manna et al., 2010), mindfulness meditation (Tomasino et al., 2014), or breath-based meditation (Wang et al., 2011) activate a frontal-parietal circuit (for a review see Cahn and Polich, 2006). These areas are related to self-referential processes, cognitive monitoring, as well as emotional and attentional processes occurring during spiritual practice (Azari et al., 2001; Newberg et al., 2001, 2003; Cahn and Polich, 2006; Manna et al., 2010; Wang et al., 2011; Tomasino et al., 2014). Previous structural MR studies even found meditation-induced plasticity (e.g., after mindfulness meditation; Hölzel et al., 2011; Lu et al., 2014; or after long-term “Western” meditation such as yoga meditation; Lazar et al., 2005; Kurth et al., 2014). These plastic changes covered brain areas associated with cognitive control, metacognitive awareness and emotional regulation, such as the ACC, insula, prefrontal areas such as the orbitofrontal cortex, temporo-parietal junction, fronto-limbic network, and default mode network structures (Lazar et al., 2005; Hölzel et al., 2011; Fox et al., 2014; Kurth et al., 2014; Lu et al., 2014).

Brain-computer interface (BCI) studies show that mindfulness meditation training significantly increased the ability to control one’s own brain activity during motor imagery (Lo et al., 2004; Tan et al., 2009; Tan L. et al., 2014). In both BCI and NF the participant’s brain signals are directly fed back to the participant in real-time. Nevertheless, these applications differ conceptually. In BCI applications, users are required to reproduce brain signals mainly by using motor imagery strategies. These signals are then classified with the aim of controlling external devices such as a computer or wheelchair (Wolpaw et al., 2002; Kropotov, 2009). Instead of reproducing the same brain activation patterns to reach high classification accuracy such as in BCI studies, in NF applications participants aim to voluntarily modulate their own brain activity to induce changes in cognition or behavior (Kropotov, 2009; Weiskopf, 2012). Tan L. et al. (2014) showed that mindfulness meditation training significantly increased the ability to control a BCI compared to control groups receiving either music training or no treatment. The trained mindful meditation behavior required both the control of cognitive process (i.e., attention on self-regulation) and monitoring the stream of consciousness (Bishop, 2004; Tan L. et al., 2014). Hence, increased skills in self-regulatory and self-awareness dependent tasks, based on increased spiritual practice—in this case mindfulness meditation training (Tan L. et al., 2014)—might be of high relevance for successful BCI as well as NF control.

Hitherto, only a few NF studies have explicitly assessed subjective verbal reports of mental strategies during NF training as indicator of mental states or processes during NF control (Angelakis et al., 2007; Nan et al., 2012; Kober et al., 2013). Self-reports showed that what is considered a useful strategy varies among individuals (Nan et al., 2012) and that mental strategies play an essential role in successful NF performance (Kober et al., 2013). For most NF training protocols, there are no clear instructions on how to modulate the target EEG parameter in the desired direction. This might explain the variety of mental strategies reported by NF users (Angelakis et al., 2007; Nan et al., 2012; Kober et al., 2013; Gruzelier, 2014a,b). Therefore, NF control can be considered as a rather underspecified task. Instructions on how to increase the sensorimotor rhythm (SMR, 12–15 Hz) voluntarily, for instance, are more prescriptive than descriptive. The SMR generally emerges when one is motionless yet remains attentive (Pfurtscheller, 1981; Sterman, 1996, 2000). While the standard instruction to increase SMR is being mentally focused and physically relaxed, verbal reports about what NF responders actually do to increase SMR range from “doing nothing in particular” to concentrating on the feedback display (Kober et al., 2013). Tasks such as NF training, which cannot be specified over a given extent by more precise and detailed instructions, are more susceptible to task-irrelevant thoughts. These thoughts might occur in an unpredictable way and can have the most diverse contents (Angelakis et al., 2007; Nan et al., 2012; Kober et al., 2013).

Importantly, people who are regularly engaged in spiritual practice, such as prayer, can be assumed to be “experts” in regulating self-referential processes and focusing their attention on internal states (Moss, 2002). Since self-awareness processes presumably are an essential part in gaining control over one’s own brain activity in NF applications (Ninaus et al., 2013, 2015), we hypothesize that people who pray frequently might show an increased ability to modulate their own brain activity during NF as compared to people who do not pray on a regular basis (Garrison et al., 2013a,b).

If frequency of praying and NF learning are somehow connected, group differences should be observable already after a very short training period of one single session. In the present study, people praying frequently and a control group of individuals praying rarely were engaged in one session of EEG based NF training, in which they should voluntarily increase SMR power derived over motor areas. The SMR rhythm is generally largest over central scalp regions covering the sensorimotor cortex. Because there is a large body of literature showing that spiritual practice, such as praying and meditating, as well as mindfulness practice, are associated with brain plasticity (Lutz et al., 2004; Lazar et al., 2005; Hölzel et al., 2011; Lu et al., 2014; Tan L.- F. et al., 2014), we also investigated possible differences in brain structure between people praying frequently and people who do not. Furthermore, we focused on the question whether the individual ability to control one’s own brain activity is related to volumetric aspects of the brain. Moreover, we investigated whether the relation between NF performance and brain structure is related to the frequency of spiritual practice, in the present case, with prayer. Additionally, we recorded subjective reports of mental strategies used during NF training to investigate a possible relationship with NF performance (Kober et al., 2013).

## Materials and Methods

### Participants

A total of 40 healthy adults (18 males and 22 females, aged 19–39 years) participated in this study. To recruit individuals who identified themselves as religious and showed a high frequency of spiritual prayer, an advertisement was distributed in spiritual/religious institutions (e.g., theological faculty of the University of Graz). Participants with average or low frequency of spiritual practice were recruited by an advertisement in the general population. All participants were naive regarding NF, gave written informed consent and were paid for their participation (7.50 € per hour). The ethics committee of the University of Graz, Austria approved all aspects of the present study in accordance to the Declaration of Helsinki. To assess the frequency of prayer, we asked the participants “*How often do you pray or meditate?*” using a five-point Likert-type scale ranging from “never” to “daily”. Half of the participants (*N* = 20, 9 males, aged 19–39 years, mean age = 24.75 years, *SE* = 1.11 years) reported to pray or meditate on a regular basis at least a few times per month. These participants were assigned to the high frequency of prayer (HF) group. The low frequency of prayer (LF) group (*N* = 20, 9 males, aged 19–35 years, mean age = 23.10 years, *SE* = 1.03 years) consisted of people who never prayed or prayed only a few times per year. Table 1 summarizes the frequency of prayer for both groups. The majority of participants of the HF group reported to be Catholics (85%), the remaining reported to be Protestants (15%). Half of the participants of the LF group also reported to be Catholics (50%), 15% reported to be Protestants and 35% seceded from Church.

**Table 1:** **Frequency of prayer of both groups as assessed with a five-point Likert-type scale**.

	Absolute number of answers	
How often do you pray or meditate?	High frequency of prayer group (HF)	Low frequency of prayer group (LF)
Never	0	17
A few times per year	0	3
A few times per month	7	0
A few times per weak	9	0
Daily	4	0

### Questionnaires

To investigate different personality traits that might be related to the frequency of prayer and NF performance, we used different questionnaires to assess spirituality and religiosity, mindfulness, LOC and control believes.

To assess the level of spirituality and religiosity, we used the Centrality of Religiosity Scale (CRS; Huber and Huber, 2012), which has a strong inter-religious conceptual base. The focus of the CRS is placed on the interactions of the centrality and content of religiosity. This scale contains 15 items. Questions are evaluated using a five-point Likert-type scale (1 = not at all, 5 = very much) and include questions such as “*How often do you think about religious issues?*”, “*To what extent do you believe that God or something divine exists?*”, or “*How important is personal prayer for you*?”. The items can be split up in five scales: intellect, ideology, public practice, private practice, and experience. Cronbach’s Alpha coefficients range from 0.72 to 0.92 (Huber and Huber, 2012). The CRS has been applied yet in more than 100 studies in sociology of religion, psychology of religion and religious studies in 25 countries with in total more than 100,000 participants (Huber and Huber, 2012).

Mindfulness was assessed using the short form of the Freiburg Mindfulness Inventory (FMI-14, Walach et al., 2006). The FMI-14 is a consistent and reliable scale validated in diverse samples (Kohls et al., 2009; Büssing et al., 2013). This questionnaire measures two different but closely related dimensions of mindfulness, which is “Presence” and “Acceptance”. It contains 14 items, which are evaluated using a four-point Likert-type scale (1 = rarely, 4 = almost always). Example items are “*I am open to the experience of the present moment*”, “*I am able to appreciate myself*”, “*I watch my feelings without getting lost in them*”, or “*I am friendly to myself when things go wrong*”. The FMI-14 shows an internal consistency of Cronbach Alpha = 0.86 (Walach et al., 2006).

LOC and control believes were assessed using the KUT questionnaire (Kontrollueberzeugung im Umgang mit Technik, i.e., control beliefs towards technology (Beier, 1999, 2004) and the FKK questionnaire (Fragebogen zu Kompetenz- und Kontrollueberzeugungen, i.e., competence and control beliefs questionnaire; Krampen, 1991). The KUT questionnaire assesses control beliefs in the context of dealing with technology (Beier, 1999, 2004). This one-dimensional construct of LOC is a subjective 5-point Likert scale rating that considers actual technologic biography in eight items (range of total score: 8–40). Example items are “*I really enjoy finding a solution for technological problems*” or “*Most of the technological problems that I have to face can be solved by myself*”. The questionnaire is available in German and has a high reliability (Cronbach Alpha between 0.74 and 0.84).

The FKK questionnaire measures three aspects of LOC and one aspect of competence orientation, namely internality, powerful others externality, chance control externality, and self-concept on four distinguishable scales. These four scales can be aggregated into two secondary scales and a tertiary scale (internality vs. externality in control beliefs). This tertiary scale was used for purposes of data analysis in this study. Example items are “*Whether I have an accident or not totally depends on my own behavior*” (internality), or “*Whether I have an accident or not strongly depends on the behavior of other people*” (externality). In sum, the FKK contains 32 items. This questionnaire was first published in German. The tertiary scale has a high reliability of Cronbach Alpha > 0.83 (Krampen, 1991).

### Description of NF Paradigm

The NF system provided audio-visual feedback for increasing SMR power (12–15 Hz) while keeping other frequencies low (theta: 4–7 Hz, beta: 21–35 Hz). EEG signal was recorded over electrode position Cz. Three vertically moving bars, depicting the power of the three feedback frequencies, were presented on a screen. The bar in the center of the screen displayed the SMR power. The bars on the left and the right side of the screen represented theta activity and beta activity, respectively (Kober et al., 2015a,b). Participants were rewarded by getting points, which were also displayed on the feedback screen, and a sound. Positive feedback was delivered when SMR power increased above an individually predefined threshold, while keeping the other two frequencies below an individually predefined threshold. A 3-min baseline/resting measurement at the beginning of the task was used to define these individual thresholds (SMR: mean of SMR power during rest, theta and beta: mean + 1 SD of theta or beta power during rest). The NF session contained nine feedback runs of 3 min each.

Individual NF performance was quantified by regression slopes of trained feedback frequencies (SMR/theta) across the nine feedback training runs. Regression slopes were estimated individually (predictor variable = feedback run number; dependent variable = *z-*transformed power of SMR/theta) and subsequently averaged per group. One-sample *t*-tests were calculated for each group to verify the existence of group effects on NF learning. Only SMR and theta were included in the analysis of the NF performance, since SMR and beta are highly correlated frequencies (Kober et al., 2015b).

After the NF training session, participants were asked to verbally describe the mental strategy they have used to gain control over the moving bars. The verbal reports were recorded electronically. Before starting NF training, participants did not receive any specific instruction on how to control the moving bars. They only got the minimal instruction of being physically relaxed and mentally focused during NF training to avoid the production of artifacts. Participants reported the usage of different strategies during NF training. The reported mental strategies were divided into 13 different categories (Nan et al., 2012; Kober et al., 2013): visual strategies, auditory strategies, cheering strategies, counting, motor activity, emotional strategy, achievement-oriented, meditation/praying, relaxation, concentration, breathing, problems with verbalization of mental strategy, no specific strategy/doing nothing. Mental strategies, which were classified as visual strategies, contained imagination of colors or objects. Auditory strategies reflected the imagery of tones or sounds. Participants using cheering strategies tried to increase the bar in the center of the screen by cheering it or themselves on. Others tried to count mentally (counting) or to tense muscles (motor activity). Participants that used emotional strategies reported that they were thinking of situations with positive or negative emotional content. Achievement-oriented participants tried to increase the number of reward points as much as possible. Some participants tried to meditate or to pray. Others tried to relax as much as possible to increase SMR. Concentration strategies refer to focused attention and concentration on the moving bars. Breathing methods were used as well, where participants tried to consciously regulate their breath to gain control over their own brain activity. Some participants had problems with verbalizing the mental strategy employed. Hence, they reported not to be able to explain in words what they have been doing during NF training. The last category included all reports in which participants explained that they tried to do nothing in particular and to just “let it happen”.

### EEG Data Recording and Analysis

EEG recording during the NF training session was performed with a g.USBamp 16 channels standard amplifier (g.tec, Graz, Austria). Vertical and horizontal EOG was recorded with three electrodes in total, two were placed on the outer canthi of the eyes and one was placed superior to the nasion. Electrode impedances were kept below 5 kΩ for the EEG recording and below 10 kΩ for the EOG recording. EEG signals were digitized at 256 Hz and filtered with a 0.5 Hz high-pass and a 60 Hz low-pass filter.

Data analysis of EEG recordings was performed offline using the Brain Vision Analyzer software (version 2.01, Brain Products GmbH, Munich, Germany). Ocular artifacts such as eye blinks or eye movements were manually rejected by visual inspection based on the information about EOG activity, provided by the EOG channels. After ocular artifact rejection, other artifacts (e.g., muscle activity) were rejected by means of a semi-automatic artifact rejection (criteria for rejection: >50 μV voltage step per sampling point, absolute voltage value >±200 μV). To analyze the NF performance, absolute values of SMR (12–15 Hz), theta (4–7 Hz) and beta (21–35 Hz) power were calculated and averaged separately for each 3-min feedback run using the Brain Vision Analyzer’s built-in method of complex demodulation. The complex demodulation is based on the complex (analytical) signal of a time series, where all frequencies except the one of interest are filtered out (Draganova and Popivanov, 1999; Brain Products GmbH, 2009). Furthermore, EEG power spectra were calculated using Fast Fourier Transformation (FFT). FFT was computed for the segmented resting measurements before the start of the NF training (segment length 2 s) with maximum resolution of ~0.50 Hz. Furthermore, a 10% Hanning window was applied including a variance correction to preserve overall power.

### Assessment of Brain Structure/Image Acquisition and Analysis

To control for the impact of measurement order on empirical results, neuroimaging data were acquired in half of the participants before the NF session and in half of the participants after the NF session (randomized order). We used a 3.0 T Siemens Skyra magnetic resonance imaging (MRI) scanner at the MRI-Lab Graz (Austria^1^). Participants were positioned in supine orientation with their head located in a 32 channel head coil. Structural images were collected using a three-dimensional T1-weighted magnetization prepared gradient-echo sequence (MPRAGE) protocol with 192 contiguous slices (TR = 1650 ms TE = 1.82 ms acquisition matrix = 256 × 256 × 192, flip angle = 8°, 1 × 1 × 1 mm voxel size, TI = 1000 ms). To minimize head movements of the participants, foam padding was used around the head within the head coil. Additionally, participants were given ear plugs to reduce discomfort due to scanner noise.

Voxel-based morphometry (VBM) analysis was used to investigate brain anatomy. Structural T1-images were processed using the VBM8 toolbox^2^ and the SPM8 software package^3^. The VBM8 toolbox provides automated gray matter segmentation routines with very high accuracy and very high reliability (Eggert et al., 2012). In a first step, structural T1-images of each participant were manually reoriented with the coordinate system’s origin set to the anterior commissure. T1 images of the participants and the default tissue probability map (VBM8) were then used as input to segment the structural images into gray matter, white matter and cerebrospinal fluid by using the default estimation options in VBM8. The resulting gray and white matter images were then normalized to Montreal Neurological Institute (MNI) standard space by using the high dimensional DARTEL (diffeomorphic anatomical registration through exponentiated lie algebra; Ashburner, 2007) approach implemented in VBM8. The resulting images were then multiplied by the non-linear components only. This approach allows comparing the absolute amount of tissue corrected for individual brain sizes and is the recommended approach in the VBM8 manual for such analyses. Afterwards the modulated and normalized images were smoothed with a Gaussian kernel of 8-mm full width at half maximum (FWHM) using SPM8.

To analyze the preprocessed data, multiple regression analyses were performed with individual NF performance (regression slopes for individual NF performance), age and gender as regressors, separately for gray and white matter in the high and low frequency of prayer group. Additionally, to check for any existing differences in gray and white matter between the two groups, a two sample *t-*test was employed. For the multiple regression analysis, voxels with a gray/white matter probability value below 0.1 were eliminated. Whole brain analysis results were corrected for multiple comparisons on cluster level with *p* < 0.05 ([family-wise error (FWE) *p* < 0.001 [voxel-level, uncorrected]). However, as the smoothness of VBM data is non-isotropic, a non-stationary correction of the data was performed. VBM data were additionally corrected for non-isotropic smoothness (Hayasaka et al., 2004) as implemented in the VBM8 toolbox^4^.

## Results

### Questionnaire Results

According to the results of the questionnaire data, the HF group showed significantly higher scores in the CRS compared to the LF group. The HF group also showed higher values in mindfulness compared to the LF group as assessed with the FMI-14. Moreover, groups were comparable in their LOC and control beliefs (Table 2).

**Table 2:** **Results (mean and SE) of questionnaires assessing spirituality (Centrality of Religiosity Scale, CRS), mindfulness (Freiburg Mindfulness Inventory, FMI-14) and control beliefs about perceived abilities to deal with technology (KUT) and the tertiary scale of the FKK (internality vs. externality in control beliefs)**.

Questionnaire	Means and SE		Results of *t*-tests	Results of correlation with NF slope (*N* =40)
	High frequency of prayer group (HF)	Low frequency of prayer group (LF)		
Overall spirituality (*z*-value)—CRS	3.72 (0.12)	1.66 (0.08)	*t*_(38)_ = 14.35, *p* < 0.01	*r* = 0.38, *p* < 0.05
Mindfulness (*z*-value)—FMI-14	0.80 (0.27)	0.05 (0.20)	*t*_(38)_ = 2.28, *p* < 0.05	*r* = 0.21, *p* = 0.20
Control beliefs (raw score)—dealing with technology—KUT	32.65 (0.79)	32.50 (0.99)	*t*_(38)_ = 0.12, *p* = 0.91	*r* = 0.08, *p* = 0.65
Control beliefs (*t*-score)—internality vs. externality—FKK	57.45 (1.33)	53.90 (1.65)	*t*_(38)_ = 1.67, *p* = 0.10	*r* = 0.18, *p* = 0.26

*Results are presented separately for the high and low frequency of prayer groups. On the right side the results of the t-tests performed for group comparisons and results of correlation analyses between neurofeedback (NF) slopes and questionnaire results across all participants are depicted*.

### NF Performance

To investigate EEG activity in the HF and LF groups during rest, we compared the absolute EEG power in different frequency bands (theta 4–7 Hz, lower alpha 7–10 Hz, upper alpha 10–12 Hz, SMR 12–15 Hz, beta 1 16–20 Hz, beta 2 21–26 Hz, and gamma 40–44 Hz power) that was recorded during the baseline interval before the NF training. All statistical comparisons were non-significant (all *p* > 0.30).

**Figure 1:**
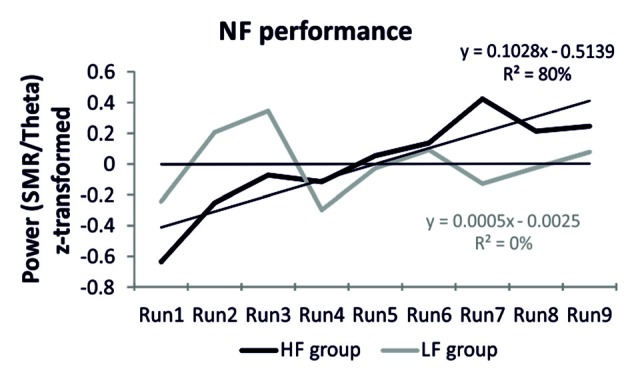
**Neurofeedback (NF) performance**. *Z*-transformed EEG power for the feedback frequency bands sensorimotor rhythm (SMR/theta) over the nine NF training runs, presented separately for the high frequency (HF) and low frequency (LF) group. Additionally, the regression equations are depicted as well as the regression lines for each group are indicated by finer black lines.

The HF group was able to voluntarily increase their SMR/theta ratio after one session of NF training (Figure 1). This was reflected in a linear increase of the power ratio in the target frequency bands. When individual SMR/theta ratio was regressed on run number 1–9, 14 out of 20 participants, reporting a high frequency of prayer (70%), were able to linearly increase their SMR/theta ratio, as suggested by positive individual regression slopes. One sample *t*-tests revealed that regression slopes in the HF group were significantly larger than zero (*t*_(19)_ = 2.82, *p* < 0.01). In contrast, the LF group was not able to linearly increase their EEG activity over the nine feedback runs within one session of NF training (Figure 1). Half of the participants of the LF group showed a positive regression slope, which indicated a linear increase in SMR/theta power across the feedback runs, while the other half showed a negative slope. Hence, the one sample *t*-tests revealed that the individual regression slopes of this group did not differ from zero (*t*_(19)_ = 0.01, *n.s*.). We also compared directly the slopes between groups. A *t*-test revealed a significant difference between the slopes of the HF and LF group (*t*_(38)_ = 1.89, *p* < 0.05; Figure 1). The NF training performance was positively correlated with spirituality as assessed with the CRS (Table 2).

### Individual Mental Strategies during Neurofeedback Learning

**Figure 2:**
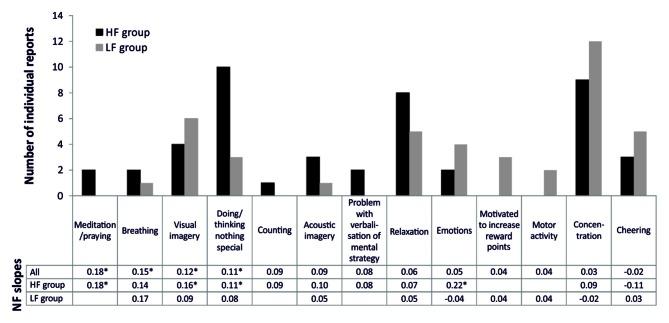
**Number of individual reports of mental strategies used during one session of SMR based NF training, presented separately for the HF and LF group**. Furthermore, we added the values of the regression slopes observed among the practitioners of each one of the reported strategies, presented separately for all participants reporting a specific strategy, the HF and LF group. Significant regression slopes are marked with asterisks (*p < 0.05).

Figure 2 depicts the frequencies of mental strategies applied during the SMR based NF training session. Note that most participants reported more than one mental strategy. When considering absolute frequencies, the HF group reported more often no specific strategy/doing nothing, relaxation, or to meditate/pray in comparison to the LF group. In contrast, the LF group more frequently reported concentration strategies, visual imagery, cheering strategies, motor activation, emotional strategies, and achievement-oriented strategies. When comparing frequencies of the reported mental strategies statistically, the HF group reported significantly more often the usage of no specific strategy/doing nothing than the LF group (${\text{χ}}_{\text{(1)}}^{\text{2}}$ = 5.58, *p* < 0.05). All other group comparisons were statistically not significant. However, the LF group tended to use cognitive effortful strategies more often, while the HF group used effortless strategies more frequently. The SMR regression slope—mental strategy associations are depicted in Figure 2, too. Meditation and praying led to the most successful NF performance, followed by breathing strategies, visual imagery and doing nothing specific. Except for visual imagery, these successful strategies were more frequently reported by HF than LF individuals.

### VBM Results

Group comparisons: two-sample *t*-test comparisons revealed no average difference between the HF and LF group regarding gray or white matter volumes.

**Figure 3:**
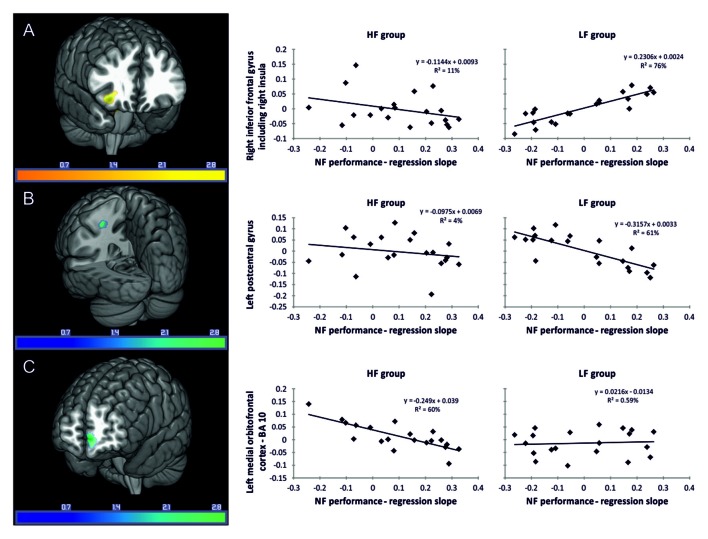
**Associations between gray matter volume (*T*-values) and individual NF performance**. Reported coordinates in montreal neurological institute (MNI) space *p* < 0.05 corrected for multiple comparisons on cluster-level [family wise error (FWE)] empirically determined extent threshold results corrected for non-stationary smoothness. **(A)** Positive association between gray matter volume and individual NF performance in the LF group. **(B)** Negative association between gray matter volume and individual NF performance in the LF group. **(C)** Negative association between gray matter volume and individual NF performance in the HF group. The right panel demonstrates the correlation between the individual NF performance and gray matter volume extracted for the regions of interest (ROIs) **(A)** right inferior frontal gyrus including the right insula, **(B)** left postcentral gyrus and **(C)** left medial orbitofrontal cortex, presented separately for the HF and LF group.

LF group: regression analysis revealed that individual NF performance was positively associated with higher gray matter volumes in the right inferior frontal gyrus including the right insula (Table 3, Figure 3A). All coordinates are reported in MNI space (Table 3, Figures 3A–C). Negative associations between gray matter volume and individual NF performance were found in gray matter volume in the left postcentral gyrus (Table 3, Figure 3B). No significant results were observed in white matter volumes.

**Table 3:** **Results of the multiple regression analysis**.

	Brodmann areas	Voxels	Peak			*T*-value (*df*, *p*-value)
			*x*	*y*	*z*	
LF group—positive associations
Orbital part of right inferior frontal gyrus including right insula	47, 34, 28, 13	681	30	25.5	−13.5	5.99 (16, 0.001)
LF group—negative associations
Left postcentral gyrus and inferior parietal lobe	40, 3, 4	225	−30	−31.5	48	5.89 (16, 0.001)
HF group—negative associations
Left medial orbitofrontal cortex	10, 11	439	−7.5	60	−3	5.84 (16, 0.001)

*Brain regions that showed associations between gray matter volume and individual NF training performance. Reported coordinates in Montreal Neurological Institute (MNI) space p < 0.05 corrected for multiple comparisons on cluster-level [family wise error (FWE)] empirically determined extent threshold results corrected for non-stationary smoothness*.

HF group: in the HF group regression analysis revealed significant negative associations between individual NF performance and gray matter volume in the left medial orbitofrontal cortex only (Table 3, Figure 3C).

## Discussion

We investigated associations between brain structure and the ability to gain control over one’s own brain activity in individuals reporting a high frequency of prayer (HF), hence considered as experts in self-awareness processes and focusing attention on internal states, and people reporting a low frequency of prayer (LF). As initially hypothesized, the HF group showed an improved ability to control their own brain activity compared to the LF group. Results of the VBM analysis revealed specific associations between brain structures and NF performance between both groups. Reports on mental strategies used during NF training also differed between groups.

Individuals who pray frequently showed a strong relationship between their individual NF performance and gray matter volume in the left medial orbitofrontal cortex, Brodmann’s area (BA) 10. Imaging studies as well as lesion studies suggest that this brain region is mainly involved in tasks in which beneficial behavioral patterns are not clearly defined (Burgess et al., 2007a; Fleming et al., 2010). This is in line with the underspecified character of the cognitive task of training SMR NF, in which task-irrelevant thoughts might occur (Angelakis et al., 2007; Nan et al., 2012; Kober et al., 2013). Successful performance in underspecified tasks requires participants to show self-initiated multitasking, to generate and maintain goals and constraints while interacting with the outside world and to concentrate and focus on external stimuli and on inner mental experiences (e.g., “on thoughts in one’s head”) at the same time (Goldstein et al., 1993; Burgess et al., 2000, 2007a,b; Goel and Grafman, 2000). According to the gateway hypothesis, BA 10 supports mechanisms operating as a “gateway” between the internal mental life that occurs independently of environmental stimuli (e.g., self-generated or maintained thoughts or representations) and the mental life that is associated with the interaction with the external world (stimulus dependent; Burgess et al., 2007a). In this context, successful EEG NF learning seems to depend on two main factors: (i) being able to focus on the feedback in real time; and (ii) being able to suppress inner mental activity detrimental to (i). While concentrating on feedback is a quite straightforward aspect of any form of NF training, monitoring or even trimming inner mental experience during NF and suppressing mental activity detrimental to focus on feedback will depend on continuous monitoring of goal-related and goal-irrelevant thoughts. Achieving this mental state depends on the ability to redirect attention away from irrelevant thoughts and towards goal-related thoughts, as well as towards the external source of feedback. In line with this, Farb et al. (2007) found that suppressing stimulus-independent thoughts (SIT) and task-irrelevant processes while focusing attention on the present moment, which is generally the case during spiritual practice, leads to a deactivation in the medial BA 10 (Farb et al., 2007). A successful suppression of NF irrelevant cognitive processes might explain the negative association between gray matter volume in BA 10 and NF performance, which we have found in individuals who pray frequently. Noteworthy, mindfulness and focusing attention on the present moment are negatively related to gray matter volume and functional activation in the left orbitofrontal cortex (BA 10; Newberg et al., 2001; Farb et al., 2007; Hölzel et al., 2011; Lu et al., 2014).

The analysis of the verbally reported mental strategies revealed differences between groups. The HF group reported more often that they did not use a specific mental strategy or that they did nothing in particular during NF control compared to the LF group. Thus, they were not able to verbalize their specific mental strategy. This might indicate that mental states during successful NF performance and mystical experience during praying or meditating are quite similar, since the latter is also related to ineffability, meaning that mystical experience is something one cannot put into words (Francis and Robbins, 2015). However, reports about doing nothing in particular does not necessarily mean that the HF group experienced this sense of ineffability/a mystical experience during NF. HF individuals also described more often the usage of relaxation strategies or meditation and prayer during NF compared to the LF group, although these group differences did not reach statistical significance. This might indicate that they did not need a lot of mental effort to focus on the NF task. In contrast, the LF group reported quantitatively more often the usage of more demanding and less effective mental strategies such as cheering strategies, motor activation, emotional strategies or achievement-oriented strategies. In line with Kober et al. (2013), more effortless cognitive strategies, such as “doing nothing special”, were overall more successful than more exhausting strategies, such as cheering (Kober et al., 2013). Generally, mental strategies reported in the present study were comparable to verbal reports described in the study by Kober et al. (2013). This indicates that there is a degree of consistency in terms of the range of strategies that people actually use during NF. Frequently reported mental strategies, such as “concentration” and “doing nothing”, are in line with the demands we put on the supervisory attentional gateway system and indicate good ability to stop or correct mind drifts, since “doing nothing in particular” and being “concentrated” are expected to have exactly this positive effect on NF performance.

We did not find any differences in brain structure between the HF and LF group, which might rule out general brain volume effects. Prior studies reporting group differences associated with spirituality often compared extreme groups, e.g., Buddhist monks who meditated several hours per day with non-meditators (Lazar et al., 2005; Hölzel et al., 2008; Kurth et al., 2014). However, there are also studies that found volumetric differences following brief 8-week or even shorter mindfulness interventions (Fox et al., 2014). Furthermore, small effects might not have been revealed by our relatively small sample size. Based on our results, we thus cannot fully exclude differences in brain structure between groups. Our correlative results indeed implicate that the left medial orbitofrontal cortex plays a different role in cognitive control processes during NF learning in both groups.

The groups did not differ regarding general or specific measurements of perceived LOC. Moreover, both groups also did not differ in their baseline EEG power in any of the frequencies employed in NF training. These findings indicate that the groups were comparable in factors previously identified as predictors of NF performance (Witte et al., 2013; Reichert et al., 2015).

In contrast to the involvement of BA 10 described so far, the LF group showed a positive association between gray matter volume in the right inferior frontal gyrus including the right insula and individual NF performance. This result supports prior findings that a network of frontal brain areas, including the insula and inferior frontal gyrus, is involved in successful NF control (Ninaus et al., 2013, 2015; Emmert et al., 2016). Ninaus et al. (2015) also used VBM analysis to investigate structural correlates of successful SMR based NF performance in healthy middle-aged adults. In the study by Ninaus et al. (2015) participants were not selected based on their frequency of prayer or any spiritual practice. Accordingly, we assume that the level of spiritual practice in the sample of Ninaus et al. (2015) is in the average range, comparable to the level of spiritual practice of the present LF group. In both studies the method to select participants was exactly the same and should lead to comparable results. Ninaus et al. (2015) found a significant relationship between volumes in the anterior insula bilaterally, left thalamus, right frontal operculum, right putamen, right middle frontal gyrus, and right lingual gyrus and the individual NF performance. Our results support and extend these findings, since we also found that the insula and the inferior frontal cortex volumes were involved in people’s ability to control their own brain activity from the very first session of NF learning. Hence, driving the attentional focus to inner states might explain the involvement of the insula (Barrett et al., 2004; Critchley et al., 2004; Pollatos et al., 2007; Craig, 2009), while top-down selection, stimulus-driven attention and maintaining attention might explain the involvement of the inferior frontal gyrus (Corbetta and Shulman, 2002; Weissman et al., 2006), both predicting the NF outcome in average individuals already during the first session of NF training. Farb et al. (2007) also found that successfully focusing attention on the present moment while suppressing stimulus independent thoughts was associated with increased activity of the right insula. The authors argued that this might be a sign that momentary self-awareness arises from the integration of basic interoceptive and exteroceptive bodily sensory processes (Farb et al., 2007).

The LF group also showed a negative association between their individual NF performance and gray matter volume in the left postcentral gyrus and left inferior parietal lobe. Ninaus et al. (2015) found a negative relation between brain volumetry in the right postcentral gyrus and NF performance. A study in experts in meditating (Theravada Buddhist monks) showed that focusing attention leads to a deactivation in the left precuneus, which might indicate self-referential activation as well (Manna et al., 2010). Furthermore, a common finding in meditation studies is that meditation leads to a decreased activation in the superior parietal lobe, probably reflecting an altered sense of space experienced during meditation (Newberg et al., 2001, 2003). Hence, a negative association between gray matter volume in parietal areas and individual NF success might be related to self-referential processes or sensory perception and integration during NF.

## Limitations and Future Directions

As mentioned before, the relatively small sample size limits the generalizability of the present results. Furthermore, group assignment based on the frequency of prayer or meditation determines the limits of generalization of the present findings to other spiritual practices, as there is a huge number of different forms of spiritual practices within different spiritual traditions. The relation between NF performance and spiritual practice has to be confirmed in future studies investigating extreme groups (long-term meditators vs. average individuals) including different forms of spiritual practices (e.g., mindfulness meditation, Buddhist meditation, relaxation or breathing based meditation). Furthermore, future studies investigating the relationship between spiritual practice and NF performance should use other feedback protocols than SMR training. This will shed light on the question whether the abilities to gate incoming information provided by the NF system and to avoid task-irrelevant thoughts, allowing focusing attention on internal states and self-awareness processes, are universal NF prerequisites or are NF protocol specific. For instance, there is evidence that gamma (>40 Hz) activity is increased in long-term meditators during meditation (Lutz et al., 2004; Hauswald et al., 2015). Hence, spiritual practice might also facilitate the ability to modulate gamma activity during NF training.

## Conclusion

Individuals with a high frequency of prayer, who are assumed to be experts in focusing attention on inner states and self-referential processes, showed an increased ability to gain control over their own brain activity during NF as compared to individuals with a low frequency of prayer. Analysis of brain volumetry revealed that plastic changes in the brain, due to spiritual practice, favor control over one’s own brain activity. Importantly, brains of both groups did not differ in volume size. Hence, plastic changes in the brain based on the frequency of spiritual practice were not related to systematic growth or shrinkage of specific brain regions. Instead, differences in brain networks might explain the disparate relationship between gray matter volume of the left medial orbitofrontal cortex (BA 10) and NF control between groups. In the HF group, BA 10 might have been better integrated in cognitive control networks necessary for NF control compared to the LF group. Results of the present study revealed that BA 10 plays an important role in NF control, expanding our knowledge about neuronal and cognitive mechanisms underlying successful NF learning (Ninaus et al., 2013, 2015; Emmert et al., 2016). The involvement of BA 10 in people praying frequently indicates that suppressing task-irrelevant activity is a crucial component of successful NF performance. This new role assigned to prefrontal brain regions might thus provide an important source explaining individual differences in the ability to learn NF.

## Author Contributions

SEK, MW, MN, CN and GW: conceived and designed the experiments. MW, MN, DW and GZ: performed the experiments. SEK, MN, KK, DW, GZ and GW: analyzed the data. SEK, KK, CN and GW: contributed reagents/materials/analysis tools. SEK, MW, MN, KK, DW, GZ, CN and GW: drafting the manuscript or revising it critically for important intellectual content. All authors approved the manuscript and agreed on publishing it.

## Conflict of Interest Statement

The authors declare that the research was conducted in the absence of any commercial or financial relationships that could be construed as a potential conflict of interest.

## Footnotes

^1^http://bioimaginggraz.at/

^2^http://dbm.neuro.uni-jena.de/vbm.html

^3^http://www.fil.ion.ucl.ac.uk/spm

^4^http://dbm.neuro.uni-jena.de/vbm.html
